# A novel extracellular vesicle isolation method based on cellulose nanofiber sheets

**DOI:** 10.20517/evcna.2025.187

**Published:** 2026-04-21

**Authors:** Yukari Nagao, Hiroaki Kajiyama, Akira Yokoi

**Affiliations:** ^1^Department of Obstetrics and Gynecology, Nagoya University Graduate School of Medicine, Nagoya 466-8550, Japan.; ^2^Institute for Advanced Research, Nagoya University, Nagoya 464-8603, Japan.

**Keywords:** Extracellular vesicle, EV sheet, cellulose nanofiber, biomarker, ovarian cancer

## Abstract

Extracellular vesicles (EVs) circulate in body fluids, carrying molecular cargo from their parent cells and exerting diverse biological functions. Consequently, they have attracted considerable attention as biomarkers for disease detection and pathophysiological understanding and have emerged as potential therapeutic targets. Although the number of clinical trials involving EVs is increasing, major challenges remain, including methodological transparency and the heterogeneity of EV subpopulations. Various EV isolation methods are commonly employed, and the primary approaches are summarized in the *MISEV2023* guidelines. Each method has advantages and disadvantages; however, most conventional approaches require relatively large liquid volumes (e.g., hundreds of microliters or more) to obtain sufficient EV yields for analysis. In recent years, novel technologies have been developed to overcome these limitations by addressing constraints related to sample volume, simplicity, and accuracy. One such innovation is the cellulose nanofiber-EV sheet, which we developed in 2023. This method enables the capture and stable storage of EVs from microvolumes of body fluids (e.g., approximately 10 µL). Two application methods are available: the attaching method, in which the EV sheet is applied to moist tissue surfaces, and the soaking method, in which the sheet is soaked into body fluids. Each method offers distinct advantages. Given their unique properties, EV sheets may contribute to biomarker analysis and facilitate new research directions across diverse fields. Continued advances in EV isolation and analytical platforms will be essential to support the safe clinical implementation of EV-based diagnostics and therapeutics.

## INTRODUCTION

Extracellular vesicles (EVs) are lipid bilayer membranes released from all living cells. EVs carry various bioactive molecules, such as proteins, nucleic acids, and lipids, that reflect the molecular status of their parent cells and play key roles in intercellular communication^[1]^. In 2007, Valadi *et al*. published a milestone paper demonstrating that mRNA and microRNA (miRNA) are transferred between cells by exosomes, highlighting the importance of exosomes in intercellular communication^[2]^. Subsequently, EV research has dramatically accelerated, the field now draws significant international interest. The International Society for Extracellular Vesicles (ISEV) was established in 2011 to advance and organize global EV research, and the term was standardized as “EVs.” Furthermore, ISEV has actively sought to standardize EVs methodologies by publishing a position paper in the *Journal of Extracellular Vesicles* that defined the minimum information requirements for EV research. This guideline is regularly updated, the latest version is “*MISEV2023*”^[3]^.

EVs are abundantly present in body fluids, including serum, ascites, urine, saliva, cerebrospinal fluid, and synovial fluid, and they also circulate systemically throughout the body. Owing to these characteristics, EVs have attracted considerable attention as potential biomarkers and therapeutic targets^[4,5]^. In the context of liquid biopsy, EVs offer particular advantages for a range of clinical application, including early diagnosis, prognosis, minimal residual disease detection, treatment selection, and treatment monitoring^[6]^. Accordingly, the number of clinical trials involving EVs has increased rapidly since 2010. Studies initially focused on diagnostics and companion diagnostics, but they have expanded since 2020 to EV-based therapeutic applications, particularly in respiratory diseases and subsequently in cancer, with human administration already underway^[7]^. Despite this progress, major challenges remain, including a lack of methodological transparency and insufficient consideration of EV heterogeneity and subpopulations^[7]^. Addressing these issues will require a deeper and more integrated understanding of EV biology. Overcoming these challenges will facilitate the development of more effective and personalized EV-based diagnostics and therapeutics, ultimately contributing to a paradigm shift toward precision medicine^[7]^. However, physician-scientists comprise only a small proportion of the EV researchers. Therefore, EV analysis tailored to the clinical setting, particularly from the perspective of surgeons involved in surgical procedures, remains largely underdeveloped. EV research conducted from the physician-scientist viewpoint could have great utility in clinical settings, highlighting the need to further develop this field.

## VARIOUS EV ISOLATION METHODS AND CHALLENGES

Various EV isolation methods have been employed since the early days of EV research, many of which remain in use currently. In line with *MISEV2023*, we summarize the primary methods for EV isolation^[3]^.

(1) Differential ultracentrifugation: the most common method for separating EVs based on centrifugal force. Advantages: widely used and well-established; capable of processing relatively large sample volumes. Limitations: time-consuming; requires specialized equipment; may co-isolate protein aggregates and other non-EV particles.

(2) Density gradient centrifugation: fractionation by sedimentation velocity using density gradient solutions in ultracentrifugation. Advantages: provides higher purity than conventional differential ultracentrifugation. Limitations: labor-intensive and time-consuming; relatively low throughput; requires specialized equipment.

(3) Size exclusion chromatography: separation by size through a bead column. Advantages: gentle method that preserves EV structure; relatively simple procedure; reduced protein contamination. Limitations: dedicated columns are required; additional concentration steps might be required.

(4) Filter-based concentration: separates EVs by size using filters. Advantages: simple and relatively fast; no specialized instrumentation required. Limitations: potential filter-clogging; possible vesicle loss or deformation.

(5) Precipitation: aggregates and precipitates EVs using high molecular-weight polymers. Advantages: easy to perform; does not require specialized instrumentation. Limitations: co-precipitates proteins and other contaminants; relatively low purity.

(6) Immunoprecipitation: captures EVs with specific surface markers using antibodies against antigens on the EV surface. Advantages: relatively high purity; enables the selective isolation of EV subpopulations expressing specific markers. Limitations: limited capture capacity; relatively high cost; marker-dependent bias.

(7) Fluid flow-based separation: separates EVs using microfluidic chips or nanostructures. Advantages: rapid processing; potential for high-precision and real-time separation. Limitations: requires specialized devices; limited throughput; may require complex device fabrication.

Different EV isolation methods can preferentially enrich distinct EV subpopulations. Consequently, the selection of isolation methods represents a critical factor in EV research, as this can substantially influence experimental outcomes, illustrating the importance of utilizing an isolation method applicable to the specific research objective. However, no single method can be guaranteed to be both necessary and sufficient necessary and sufficient; in some cases, validation through comparison or combination of multiple methods is required to ensure robustness. Although rigorous evaluation of isolated EVs is critical for clinical application, standardized evaluation strategies have not yet been fully established, representing a significant challenge. Each isolation method has advantages and disadvantages, but most conventional approaches are generally limited by the requirement for liquid volumes in the hundreds of microliters to milliliters to obtain sufficient EV yields for downstream analyses^[8]^, which has restricted the types of samples available for analysis. In addition to improving the simplicity and accuracy of EV isolation, this limitation has driven the need for technological innovations capable of analyzing EVs from trace volumes of body fluids.

Currently, engineering researchers in academia and industry are developing new EV isolation methods. This section introduces several notable new technologies published in recent years.

(1) Electrokinetic deterministic lateral displacement by Gillams *et al*.^[9]^: size-dependent fractionation of nanoscale vesicles using a microfluidic pillar array device coupled with an electric-field-induced dielectrophoretic effect.

(2) Viscoelastic microfluidic EV isolation by Meng *et al*.^[10]^: a label-free technology for separating EVs based on size-dependent particle migration in viscoelastic fluids.

(3) Digital microfluid EV isolation by Tong *et al*.^[11]^: digital microfluidic technology for rapid and automated EV isolation and EV-miRNA preparation.

(4) Acoustofluidic EV isolation by Naquin *et al*.^[12]^ and Gerlt *et al*.^[13]^: acoustofluidic platforms enabling rapid EV separation and concentration using acoustic radiation forces.

(5) Flocculation via orbital acoustic trapping by Rufo *et al*.^[14]^: an acoustofluidic method that combines acoustic trapping with thermoresponsive polymer-induced flocculation.

(6) Nanowire-based EV capture and analysis by Chattrairat *et al*.^[15]^: an integrated nanowire based well-plate assay system that enables charge-based EV capture and membrane protein analysis.

(7) Cellulose nanofiber (CNF)-EV sheet by Yokoi *et al*.^[16]^: as described later.

Continued advances in these technologies, alongside expanded validation across multiple disciplines and applications, are expected in the future.

## DEVELOPMENT OF CNF-EV SHEETS

**Figure 1:**
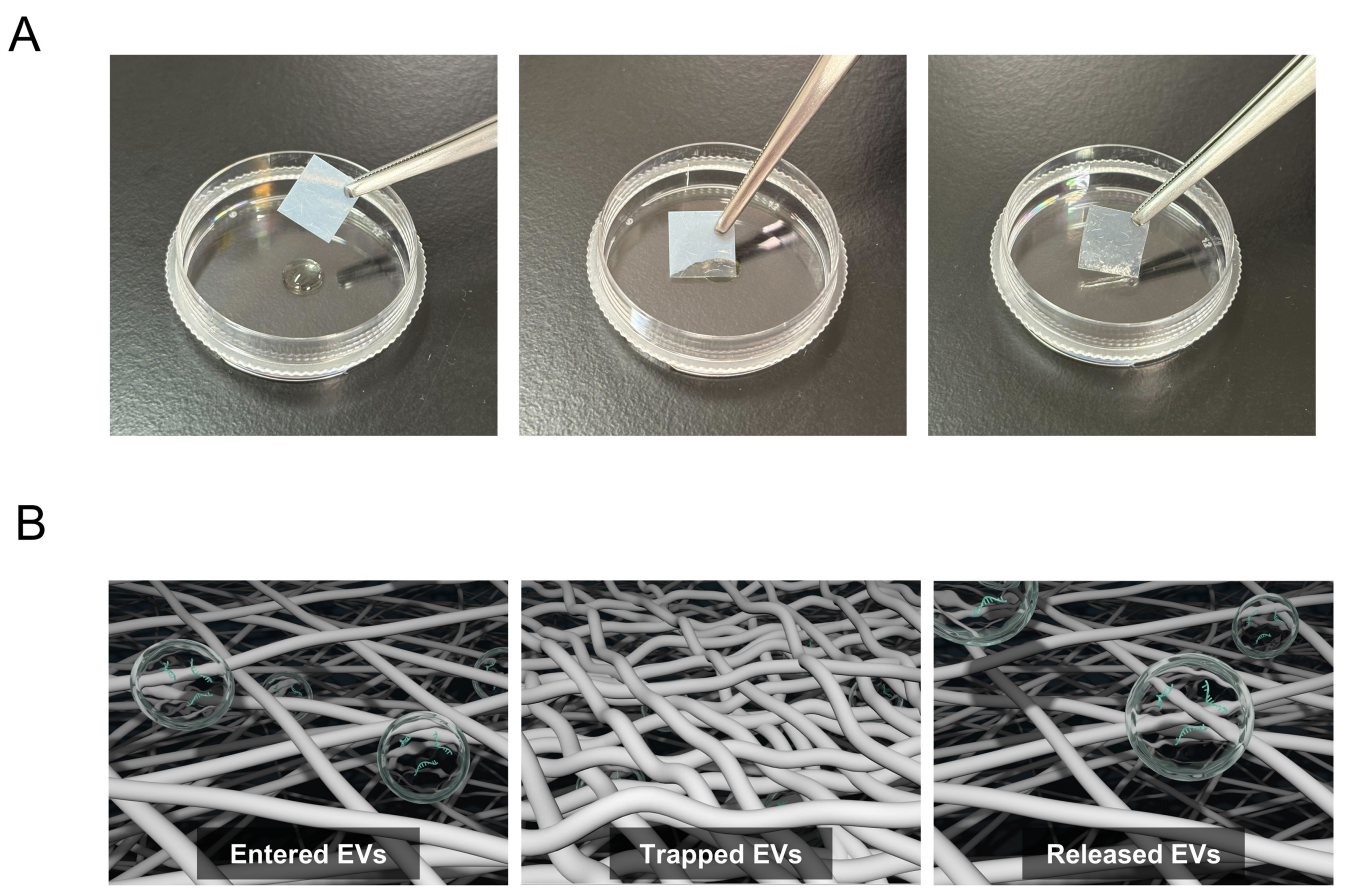
EV sheet, a cellulose nanofiber-based EV isolation platform. (A) Representative images of EV sheets prepared as approximately 1 cm^2^ square pieces. The sheets appear milky white in the dry state and become semitransparent upon water absorption. Left: dry state; Center: absorption in progress; Right: fully water-absorbed state; (B) Schematic of EV capture and release in EV sheets. Each sheet contains many small pores (< 300 nm in diameter) formed by fine cellulose nanofibers. Left: EVs are captured within the sheet. Center: Pore closure during drying retains the EVs within the pores of the sheet. Right: Soaking in PBS reopens the pores and enables the release of the retained EVs. Modified from Reference 16 based on CC BY 4.0^[16]^. EV: Extracellular vesicle; PBS: phosphate-buffered saline; CNF: cellulose nanofiber.

Against this background, our group developed a specialized paper, which we termed the EV sheet, in 2023 as an innovative EV isolation modality based on CNF [Figure 1A]^[16]^. CNF is a novel Japanese biomass material made from pulp derived from unused wood, thinning residues, and wood chips, resulting in a low environmental burden^[17]^. CNF has been widely researched, and it has attracted significant attention across multiple fields, including the automotive, building material, and home appliance sectors^[18]^. Our primary reason for selecting CNF was its shared polysaccharide backbone with surgical gauze, a feature we considered advantageous for clinical handling.

**Figure 2:**
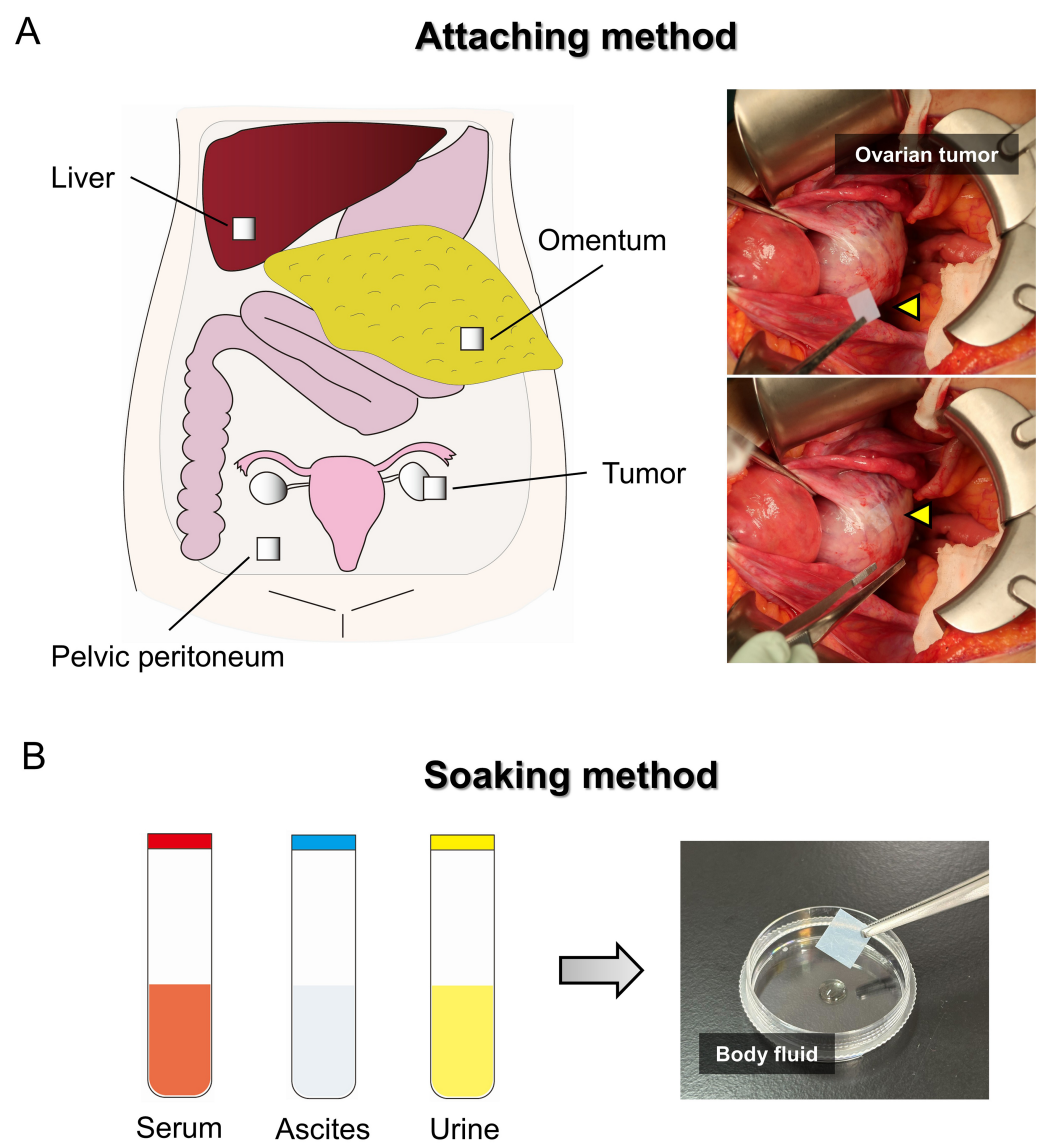
EV sheet attaching and soaking methods. (A) Schematic overview of EV collection sites within the peritoneal cavity using the attaching method, together with representative images of EV sheets attached to the ovarian tumor surface. The arrowhead indicates an EV sheet; (B) Schematic representation of the soaking method, in which EV sheets are soaked in a small volume of body fluid, such as serum, ascites, and urine. EV: Extracellular vesicle.

CNF-EV sheets are composed of fine CNFs that form numerous small pores. When the sheet contacts aqueous samples, its water-absorbing properties allow EVs to enter the porous structure, resulting in EVs retention (Figure 1B, left). During the subsequent one-week drying process, fiber aggregation leads to pore closure, effectively entrapping the EVs inside the sheet (Figure 1B, center). For EV recovery, the dried sheet is washed and immersed in PBS, which reopens the pores and permits release of the captured EVs (Figure 1B, right). The pore size distributions of EV sheets varied with each procedural step: < 300 nm at fabrication, < 10 nm after drying, < 20 nm after washing, and < 300 nm after EV recovery. Therefore, EVs collected by CNF-EV sheets are primarily CD63-positive small EVs (sEVs) with diameters of 40-200 nm. The sheets are prepared as approximately 1 cm^2^ square pieces, appearing milky white in the dry state and becoming semitransparent upon water absorption [Figure 1A]. The key advantage of this platform is its ability to efficiently capture EVs from extremely small volumes (approximately 10 µL) of body fluid. In addition, EV sheets enable stable long-term storage in a dry state at room temperature and straightforward extraction after storage. We confirmed that sEVs can be stored for at least 90 days in dry CNF-EV sheets. The basic characteristics of EVs isolated using EV sheets, including particle morphology, size distribution, expression of surface markers, and purity, are comparable to those obtained with conventional isolation methods, supporting subsequent functional analyses, including EV-miRNA sequencing. For this purpose, CNF-EV sheets dried for 7 days resulted in higher read count and annotation ratio than those dried for 1 day; thus, subsequent experiments should fundamentally be conducted using samples stored for approximately 1 week. For practical use, EV sheets can be applied in two distinct ways, the attaching method and the soaking method, as discussed in the following section [Figure 2]. Each method has its preferred applications and represents a valid, independent approach.

## APPLICATION OF CNF-EV SHEETS

### Ovarian cancer

Ovarian cancer lacks established early screening methods similar to those used for breast or cervical cancer. Furthermore, this malignancy is prone to early peritoneal metastasis, often leading to diagnosis at advanced stages^[19]^. High-grade serous carcinoma is the most common histological type, accounting for more than half of all ovarian cancers^[20]^. Accordingly, there is an urgent need to establish reliable biomarkers for early detection and recurrence prediction to improve patient outcomes. In parallel, elucidating the biological functions of specific EV populations involved in ovarian cancer progression. Additionally, the contributions of EVs derived from key peritoneal seeding sites to metastatic processes warrant further investigation.

### EV sheet attaching method

We developed the attaching method, in which an EV sheet is briefly applied to a target site, such as a moist organ surface, enabling direct EV capture from small amounts of ascites on the tissue surface [Figure 2A]. This approach represents an unprecedented strategy for the site-specific capture of individual EVs. Our previous study^[16]^ applied sterilized EV sheets intraoperatively to various intraabdominal tissues, including liver, omentum, pelvic peritoneum, and tumor, in patients with ovarian tumors. EVs collected from the sheets were subsequently isolated and subjected to miRNA sequencing.

The study revealed that EVs collected from tumor surfaces using EV sheets exhibited miRNA expression profiles that more closely resembled those of the corresponding tumor tissues than did EVs isolated from whole ascites, indicating that EV sheets can directly capture tumor-derived EVs. In patients with early-stage ovarian cancer localized to the ovaries, EV-miRNA profiles obtained from the surfaces of the liver, omentum, and pelvic peritoneum formed site-specific clusters that were distinct from the tumor tissue profile. Conversely, in patients with advanced-stage ovarian cancer involving tumor rupture or peritoneal seeding, the EV-miRNA profiles from these organ surfaces converged toward that of the tumor tissue. These findings indicate that EV-miRNA distributions within the peritoneal cavity exhibit location-dependent patterns that reflect the clinical disease status. Furthermore, analysis of miRNAs upregulated in ovarian cancer tissue revealed elevated expression of these miRNAs in EVs derived from tumor surfaces and rupture sites, with their expression gradually decreasing with increasing distance from the primary tumor.

### EV sheet soaking method

The soaking method is a simple EV isolation approach in which EV sheets are soaked in a small volume of body fluid for a few seconds [Figure 2B]. Unlike the attaching method, the soaking method captures EVs distributed in the bulk liquid. Therefore, the EV populations collected using the two methods might differ. In contrast to conventional isolation methods, which typically require 1-2 h and carry limitations in EV storage^[21-24]^, the soaking method enables EV isolation within approximately 10 min. It also allows the sheets to be stored and transported in a dry state at room temperature before extraction. Although convenient isolation and storage are important, the ability to analyze very small sample volumes is the most critical feature, as this substantially reduces invasiveness and broadens applicability, making this approach particularly valuable for specimens that are available only in limited quantities or are otherwise difficult to obtain.

In our previous study^[16]^, we identified miRNAs that were highly expressed in preoperative serum and urine and subsequently demonstrated a postoperative decline in their expression levels. These miRNAs were also highly expressed in ovarian cancer tissue, and their expression could be reliably validated by quantitative PCR. While such miRNA profiling is a common approach in cancer biomarker discovery, our EV sheet-based method is significantly simpler than conventional techniques. It also enables analysis using minimal amounts of body fluids, highlighting its practical advantages. Thus, the soaking method represents a practical and scalable strategy with strong potential for downstream clinical and societal implementation.

## FUTURE PROSPECTS

The biological significance and translational potential of EVs are widely recognized, making them a focus of active research globally. Nevertheless, major challenges related to EV isolation and storage persist, hindering the efficient translation of EV research into practical and commercial applications. Under these circumstances, EV sheets represent a platform that could fundamentally transform both EV research and clinical practice by converting challenging EV samples into readily analyzable targets. EV sheets have been introduced by the ISEV as a notable new EV isolation technology with anticipated future development^[25]^.

However, EV sheets have three primary limitations. First, although surface EVs and EVs in fluid can be collected, EVs in deeper locations, such as the extracellular matrix, might not be sufficiently collected. Second, due to the novelty of the technology, standardization for quantification remains insufficient. Third, the collection efficiency has not been clear, and specific populations susceptible to adsorption based on particle size or surface charge could exist.

**Figure 3:**
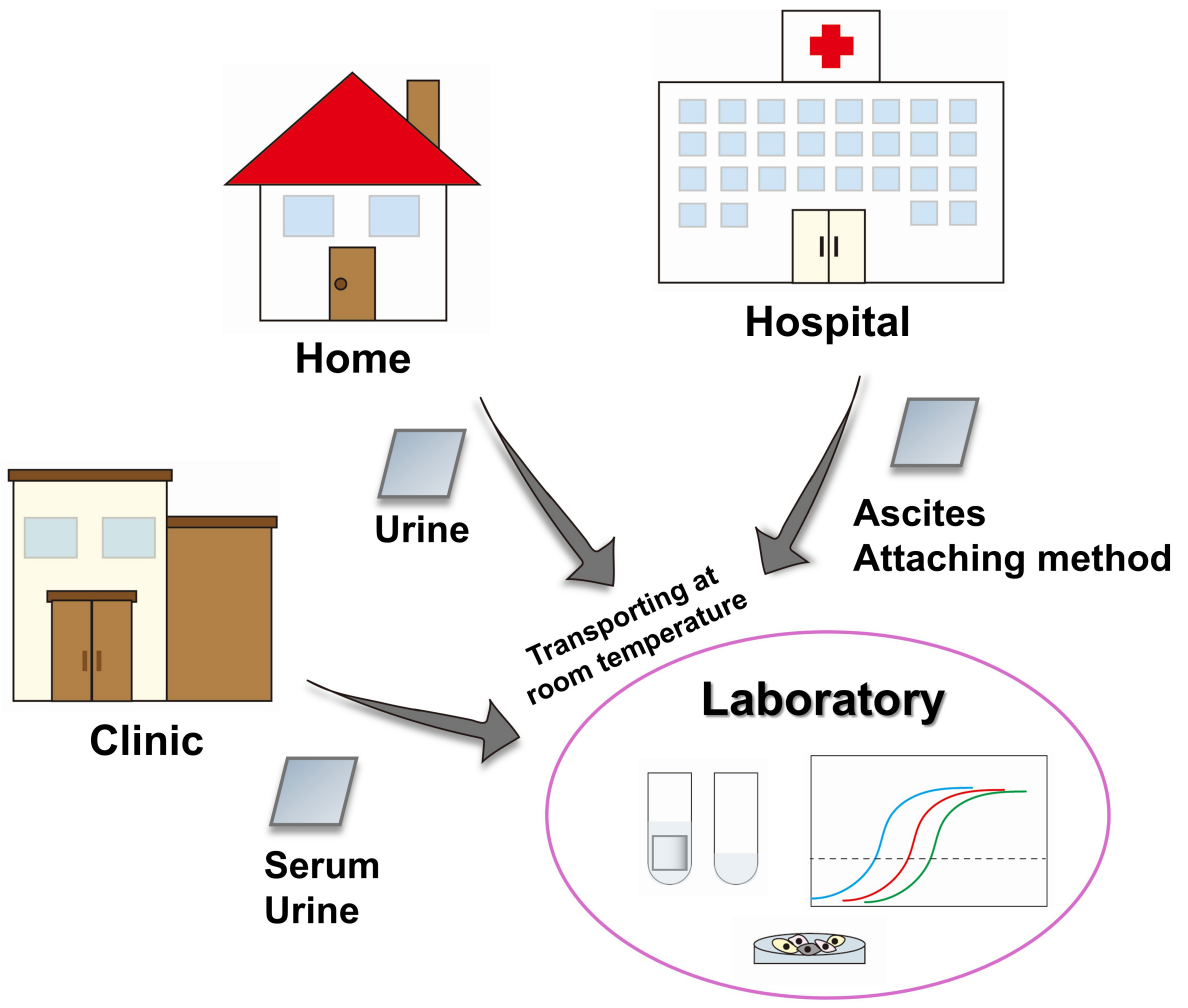
Flowchart for EV sheet clinical and societal implementation. Proposed workflow for EV collection and storage using EV sheets. EVs are collected from body fluids at home or in clinics and transported to laboratories at room temperature. In hospitals, the attaching method can additionally be applied. EV: Extracellular vesicle.

In ovarian cancer, our immediate goal is to leverage EV sheets as a platform for biomarker discovery across multiple clinical contexts, including early diagnosis, staging, treatment response prediction, and recurrence monitoring. Based on the three key advantages of EV sheets, namely simple isolation, stable storage, and transport compatibility, we envision a platform in which EVs can be directly collected from body fluids at home or in clinics using the soaking method, followed by transport to laboratories at room temperature [Figure 3]. This approach has strong potential for real world clinical implementation. When combined with the attaching method, the EV sheet platform bridges conventional molecular analysis with spatially informed intraoperative sampling, thereby extending EV research from systemic biomarkers to site-specific disease biology. Given the poor prognosis of ovarian cancer and the absence of effective population-based screening strategies, the successful establishment of this platform could provide both clinical benefits and broader real-world impact. Importantly, spatially resolved EV information obtained through the attaching method enables direct investigation of peritoneal seeding dynamics from a previously inaccessible perspective, offering new insights into the mechanisms underlying ovarian cancer progression.

In addition to their application to other cancers, EV sheets could emerge as a universal analytical platform extending beyond oncology. The applicability of EV sheets to microvolume samples could expand the scope of EV research to experimental settings involving limited biological material, including animal models and small cell populations. By enabling the analysis of EV-derived information from sample volumes that were previously impractical to study, EV sheet technology has the potential to reshape fundamental EV research and promote cross-disciplinary collaboration in EV biology.

## CONCLUSION

EV isolation technology has made significant progress, but several challenges remain, including methodological transparency, insufficient consideration of EV heterogeneity and subpopulations, and a lack of standardized quantification. Overcoming these challenges is critical for the clinical application of EV-based diagnostic and therapeutic technologies. In the future, interdisciplinary research will likely lead to the development of more robust EV analysis platforms.

## DECLARATIONS

### AI and AI-assisted tools statement

During the preparation of this manuscript, the AI tool ChatGPT (including GPT-4– and GPT-5–series models) was used solely for enhancing manuscript readability and language quality. The tool did not influence the study design, data collection, analysis, interpretation, or the scientific content of the work. All authors take full responsibility for the accuracy, integrity, and final content of the manuscript.

### Copyright

© The Author(s) 2026.
